# Simvastatin Increases Fibulin-2 Expression in Human Coronary Artery Smooth Muscle Cells via RhoA/Rho-Kinase Signaling Pathway Inhibition

**DOI:** 10.1371/journal.pone.0133875

**Published:** 2015-07-24

**Authors:** Noemí Serra, Roser Rosales, Lluís Masana, Joan-Carles Vallvé

**Affiliations:** Facultat de Medicina, Unitat de Recerca en Lípids i Arteriosclerosi, Universitat Rovira i Virgili, CIBER de Diabetes y Enfermedades Metabólicas Asociadas (CIBERDEM), Institut Investigació Sanitària Pere Virgili (IISPV), Reus, Catalonia, Spain; IIBB-CSIC-IDIBAPS, SPAIN

## Abstract

The composition and structure of the extracellular matrix (ECM) in the vascular wall and in the atherosclerotic plaque are important factors that determine plaque stability. Statins can stabilize atherosclerotic plaques by modulating ECM protein expression. Fibulins are important components of the ECM. We evaluated the in vitro effect of simvastatin on the expression of fibulin-1, -2, -4 and -5 in human coronary artery smooth muscle cells (SMCs) and the mechanisms involved. Cells were incubated with simvastatin (0.05–1 μM), mevalonate (100 and 200 μM), geranylgeranyl pyrophosphate (GGPP) (15 μM), farnesyl pyrophosphate (FPP) (15 μM), the Rho kinase (ROCK) inhibitor Y-27632 (15 and 20 μM), the Rac-1 inhibitor (another member of Rho family) NSC23766 (100 μM), arachidonic acid (a RhoA/ROCK activator, 25–100 μM) and other fatty acids that are not activators of RhoA/ROCK (25–100 μM). Gene expression was analyzed by quantitative real-time PCR, and fibulin protein levels were analyzed by western blotting and ELISA. Simvastatin induced a significant increase in mRNA and protein levels of fibulin-2 at 24 hours of incubation (p<0.05), but it did not affect fibulin-1, -4, and -5 expression. Mevalonate and GGPP were able to reverse simvastatin’s effect, while FPP did not. In addition, Y-27632, but not NSC23766, significantly increased fibulin-2 expression. Furthermore, activation of the RhoA/ROCK pathway with arachidonic acid decreased fibulin-2 mRNA. Simvastatin increased mRNA levels and protein expression of the ECM protein fibulin-2 through a RhoA and Rho-Kinase-mediated pathway. This increase could affect the composition and structure of the ECM.

## Introduction

Atherosclerosis, the primary underlying cause of cardiovascular diseases, is a systemic disease of the arterial wall that leads to plaque development[1, 2]. During the progression of atherosclerosis, the structure, abundance, and composition of the arterial wall extracellular matrix (ECM) are deeply affected[3]. Moreover, the progression of plaque can lead to a so-called vulnerable-type plaque, characterized by a thin fibrous cap and intraplaque neovascularization and hemorrhage[4, 5] among other factors. The breakdown of ECM components (collagen, elastin, and others) by extracellular proteases in atherosclerotic plaques promotes fibrous cap thinning and destabilization[6, 7], which has been associated with major adverse clinical outcomes[8], such as myocardial infarction and stroke[4, 9, 10]. Intraplaque neovascularization is characterized by new immature and thin-walled micro-vessels derived from the adventitial vasa vasorum. The consequence of this reduced wall structure is a fragile network of new vessels that can easily rupture, causing intraplaque hemorrhage[11]. Furthermore, an increased density of these immature micro-vessels has been identified at the shoulders of atherosclerotic lesions where rupture is more frequently described[12–14].

Fibulins are a family of seven proteins that are important components of the ECM[15, 16], basement membranes[17] and elastic matrix fibers[18]. Exhibiting an extensive array of protein–protein interactions, fibulins act as intermolecular bridges between ECM components, connecting various supramolecular structures. Fibulins participate in the correct assembly of elastin and microfibrils to form elastic fibers. For instance, fibulin-1 and -2 bind to tropoelastin and proteoglycans, and fibulin-5 binds to tropoelastin and elastin fibers[19, 20]. Moreover, fibulin-2 binding to fibronectin and collagen in the basement membrane has been described[21]. Therefore, fibulins have an important structural function in the arterial wall and in different types of connective tissues. The dysregulation of certain fibulins occurs in a range of human disorders[22–27]. Moreover fibulin-2 has been shown to colocalize with versican and hyaluronan in in murine vascular atherosclerotic lesions[28].

Statins comprise a class of hypocholesterolemic agents used in people with or at risk for cardiovascular disease. They lower cholesterol by inhibiting HMG-CoA reductase, but in addition, statins have a wide range of pleiotropic effects[29–31], including the stabilization of atherosclerotic plaques[32, 33]. In this regard, it has been shown that statin therapy in animal models modifies the biology of the atherosclerotic plaque and increases its stability[34]. Moreover, collagen and fibrotic content of plaques significantly increases in patients receiving statin treatment, conferring resistance to rupture and plaque stabilization[35–39]. Some of these non-lipid related actions may be explained by the inhibition of several intracellular pathways, including kinases and small G proteins [30, 40, 41]. Statins inhibit posttranslational modifications of GTPases such as RhoA and Rac1 through the inhibition of isoprenoid intermediates of the cholesterol pathway, such as farnesyl pyrophosphate (FPP) and geranylgeranyl pyrophosphate (GGPP) [40]. RhoA function is regulated through the activation of the Rho-kinase (ROCK) pathway, which is also inhibited by statins and can be activated by arachidonic acid (AA), a polyunsaturated omega-6 fatty acid[42, 43], but not by other types of fatty acids.

The effect of statins on the expression of fibulin family members has not yet been examined. Therefore, the aim of our study was to evaluate whether simvastatin could modify the expression of 4 ECM fibulin family (fibulin-1, -2, -4, and -5) in human coronary artery smooth muscle cells (SMCs). Our results indicate that simvastatin increases the expression of fibulin-2 in human coronary artery SMCs through a RhoA/ROCK-dependent mechanism.

## Methods

### Reagents

Medium 231, smooth muscle growth supplement (SMGS) and gentamicin/amphotericin B solution were purchased from Cascade Biologics (Madrid, Spain). Simvastatin sodium salt was purchased from Calbiochem (Darmstadt, Germany). GGPP, FPP and fatty acids [palmitic (PA), oleic (OA), linoleic (LA), AA, eicosapentaenoic (EPA) and docosahexaenoic (DHA)] were purchased from Sigma-Aldrich (Madrid, Spain). Y-27632 dihydrochloride and NSC 23766 were purchased from Tocris Bioscience (Bristol, United Kingdom). Dimethyl sulfoxide (DMSO) was purchased from Merck (Darmstadt, Germany).

### Human coronary artery smooth muscle cell culture

Three different lots of human coronary artery SMCs were purchased from Cascade Biologics (4C0915, 4C1284, and 886619). The donor for lot number 4C0915 was a 19-year-old male; for lot number 4C1284, a 21-year-old male; and for lot number 886619, a 36-year-old woman. The cells were cultured in medium 231 (HEPES + bicarbonate) supplemented with SMGS (fetal bovine serum (4.9% v/v), human fibroblast grow factor (2 ng/ml), human epidermal grow factor (0.5 ng/ml), heparin (5 ng/ml), insulin (5 μg/ml) and bovine serum albumin (0.2 μg/ml)) and gentamicin/amphotericin B solution at a density of 2500 cells/cm^2^ according to the manufacturer’s protocol. Cells were grown at 37°C under a humidified atmosphere and 5% CO_2_. The experiments were performed when cells had grown to 80–90% confluence. The experiments were reproduced using the three different lots of cells. The lot of cells used in each experiment is stated in the Figure Legend of each Figure.

### Effect of simvastatin on fibulins

Human coronary artery SMCs were incubated with a wide range of concentrations of simvastatin (0.05, 0.1, 0.5, 1 μM) for 6 or 24 hours. Depending on the objective of the experiments, cells were used for either total RNA or protein extraction. Cells incubated with vehicle alone (untreated cells) were designated as control.

### Effect of mevalonate, GGPP, FPP, NCS 23766 and Y-27632 on fibulin-2 mRNA expression

Human coronary artery SMCs were pre-incubated for 2 hours with mevalonate (100 and 200 μM), GGPP (15 μM), and FPP (15 μM), and with the Rho kinase inhibitor Y-27632 (10, 15, 20 μM), and the Rac-1 inhibitor (another member of Rho family) NSC23766 (100 μM) for 24 hours. After incubation, cells were used for total RNA isolation. Cells incubated with vehicle alone (untreated cells) were designated as control. In some cases the culture media was kept at -80°C for fibulin 2 protein determination.

### Preparation of Fatty acid (FA) sodium salt and FA-BSA complex

Preparation was made with some modifications according to the method of Wu et al.[44] Ten milligrams of each FA were mixed with 0.5 ml EtOH and 5 M NaOH in a 1:1 ratio volume of FA to NaOH. The mixture was dried under nitrogen gas until the FA sodium salt formed. The salt was then dissolved in 2 ml sterile water (stock solutions of FA). To avoid FA oxidation, 1 μM BHT was added to the FA stock solutions. Stock solutions of FA complexed to BSA were made by mixing FA and 5 mM BSA in a 3:1 ratio of FA to BSA. The FA-BSA solution was sterile-filtered and used fresh.

### Effect of FA-BSA on fibulin-2 mRNA expression

Human coronary artery SMCs were incubated with FA-BSA at 25, 50 or 100 μM for 24 h. The fatty acids used were PA, OA, LA, AA, EPA and DHA. Cells were used for total RNA extraction. Cells incubated with vehicle alone (untreated cells) were designated as control.

### Cytotoxicity

The potential cytotoxic effect of all compounds on human coronary artery SMCs was assessed by lactate dehydrogenase (LDH) release in the culture medium using the CytoTox 96 Non-Radioactive Cytotoxicity Assay (Promega; Madrid, Spain) following the manufacturer’s instructions and by observing cellular morphology (Olympus IX71; Barcelona, Spain). We did not observe any cytotoxic effect of the compounds used at the concentrations and times described.

### RNA isolation

Cells were lysed in lysis buffer, and total RNA was isolated from the cells using the ABI PRISM 6100 Nucleic Acid PrepStation (Life Technologies; Madrid, Spain) following the manufacturer’s instructions. The absorbance at 260 nm was used to measure the RNA concentration, and the 260/280 nm absorbance ratio was used to analyze RNA quality.

### Quantitative real-time polymerase chain reaction

A total of 0.5 μg RNA was reverse transcribed to cDNA using Random Hexamers and SuperScript II (Life Technologies) following the manufacturer’s protocol. Taqman primers and probes for fibulin-1, -2, -4 and -5, glyceraldehyde 3-phosphate dehydrogenase (GAPDH) and 18s were obtained from validated and pre-designed Gene Expression Assays (Life Technologies) and were used in real-time polymerase chain reaction (rtPCR) amplifications. mRNA expression for each gene and sample was calculated using the recommended 2^-ΔΔCt^ method. The control group (untreated cells) was defined as the calibrator in this experiment. GAPDH and 18s were used as housekeeping genes to normalize the results of the gene of interest.

### Fibulin protein extraction and quantification

The culture plates were placed on ice and the medium was removed and kept at -80°C. Next, the plates were washed with phosphate buffered saline (PBS). Then, 800 μl of PBS was added, and the cells were scraped from the surface of the culture plates with a cell filter and centrifuged at 1500 rpm for five minutes at 4°C. The supernatant was discarded. A total of 100 μl hypotonic buffer was added, and the samples were passed through a syringe with a 26½ G needle. Next, the samples were centrifuged at 16,000 x g for 10 minutes at 4°C. The supernatant, now containing cytoplasmic extracts, was stored at -80°C. For protein quantification, a Qubit Fluorometer (Life Technologies) was used.

Secreted fibulin-2 in the cell medium was determined by commercial ELISA kits (Uscn Life Science, Inc.), according to the instructions.

### Western blotting

Human coronary artery SMCs were cultured as previously described and incubated with simvastatin at concentrations of 0.05–1 μM for 6 or 24 hours. For fibulin-1 and fibulin-2, cell extracts were denatured in 20X Tris-Acetate SDS Running Buffer, separated on Novex 3–8% Tris-Acetate gels (Life Technologies) and transferred to nitrocellulose membranes (0.45 μM). This procedure was also followed for fibulin-2 determination in the cell medium prior to concentration of the samples with Amicon 10K centrifugal filters (Millipore). For fibulin-5, cell extracts were denatured in 20X MOPS SDS Running Buffer, separated on Novex 10% Bis-Tris gels (Life Technologies) and transferred to nitrocellulose membranes (0.22 μM). The membranes were blocked in 1X Tris-buffered saline (TBS), 0.1% Tween and 4% ECL advance (Western blotting detection kit (GE Healthcare; Barcelona, Spain)) for 1 hour, washed in 1X TBS and incubated with fibulin-1 and -5, actin (1:500) (Santa Cruz Biotechnology; Heidelberg, Germany) or fibulin-2 (1:500) (Novus biologics; Cambridge, United Kingdom) antibodies overnight at 4°C with continuous shaking. After further washing in 10% 1x TBS, 1% SDS and 0.5% Nonidet P-40, the blots were incubated for 30 minutes with secondary anti-goat P-0049 antibodies (1:10,000) (DACO; Barcelona, Spain) for fibulin-1, fibulin-5, and actin and with secondary anti-rabbit P-0048 antibodies (1:10,000) (DACO) for fibulin-2. Bands were visualized using ECL reagents (Life Technologies) with Versadoc (Bio Rad; Barcelona, Spain), normalized to actin expression and quantified with Quantity One Analysis Software version 4.6.2.

### Statistical analysis

All of the results are expressed as the Mean ± Standard Error of the Mean (SEM). The number of independent experiments conducted for each result is stated in the Figure Legends. Statistical analyses were performed using one-way analysis of variance (ANOVA), followed by Dunett post-test correction for multiple comparisons. Differences were considered significant at p<0.05. Statistical analyses were performed using GraphPad Prism software (San Diego, California, USA) version 5.01.

## Results

### Effect of simvastatin on fibulin expression

**Fig 1:**
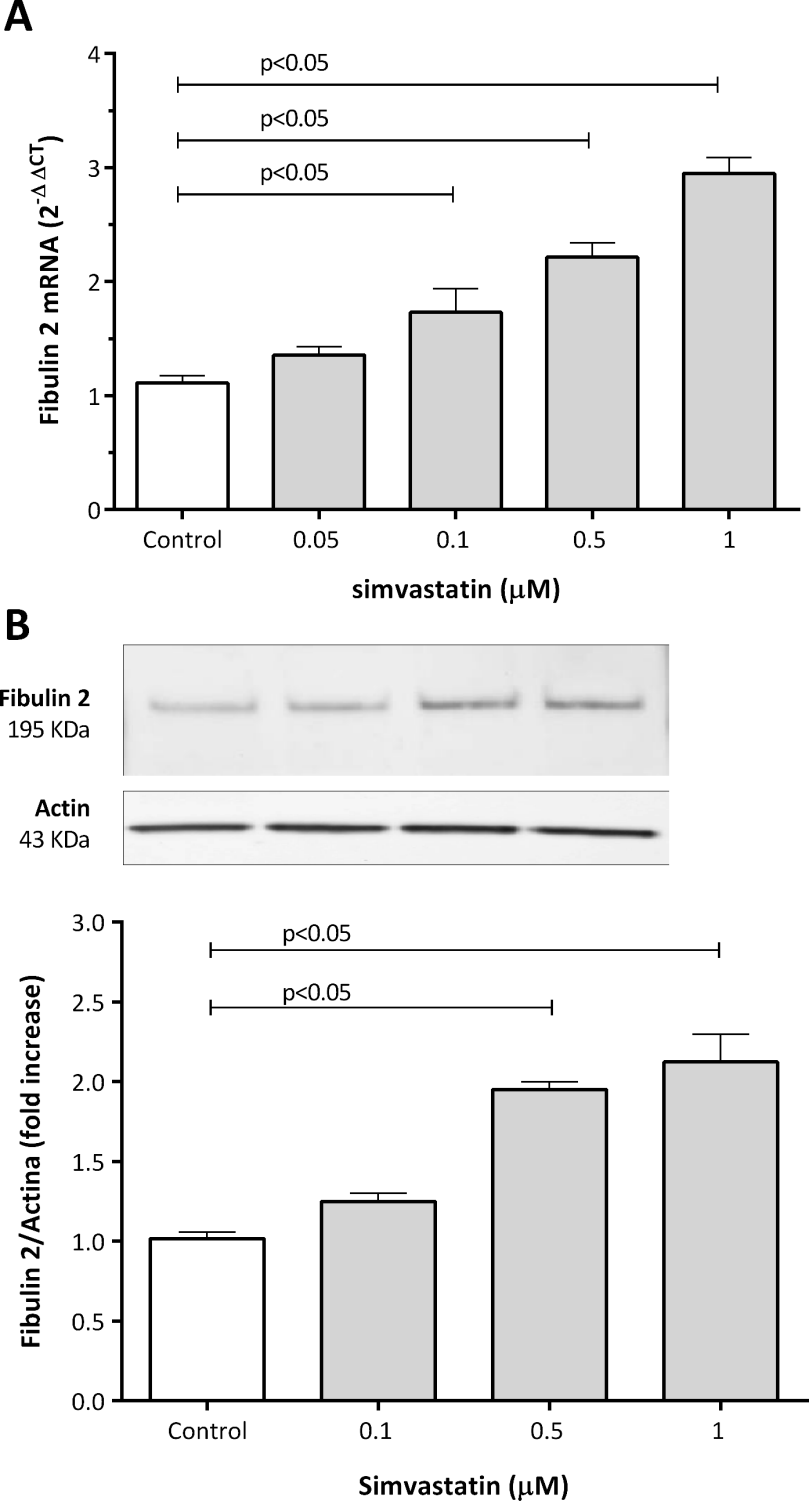
Effect of simvastatin on fibulin-2 mRNA (A) and protein (B) levels in human coronary artery SMCs. Cells were treated with simvastatin for 24 hours. Blot bands were normalized to actin and quantified with Quantity One Analysis Software version 4.6.2. A representative Western blot is shown. The results are shown as the mean with the standard error of the mean (SEM) for eight independent experiments in 1A (2 with cell lot number 4C0915, 3 with cell lot number 4C1284, and 3 with cell lot number 886619) and six independent experiments in 1B (2 with cell lot number 4C0915, 2 with cell lot number 4C1284, and 2 with cell lot number 886619). Comparisons were performed using ANOVA followed by Dunett post-test correction.

Six hours after simvastatin treatment, fibulin-1, -2, -4, and -5 mRNA levels were unchanged (S1 Fig). However, after 24 hours of treatment, simvastatin produced a significant increase in fibulin-2 mRNA levels in human coronary artery SMCs compared to controls (Fig 1). The lowest simvastatin concentration showing a significant effect was 0.1 μM with a 1.7-fold increase in fibulin 2 mRNA levels. The maximum effect of simvastatin was at 1 μM with a 2.9-fold increase. In addition to increasing fibulin-2 mRNA expression, simvastatin treatment also increased fibulin-2 protein expression. We quantified western blot band intensity and normalized them to actin expression and found a significant increase with a maximum effect also at a concentration of 1 μM with a 2.1-fold increase (Fig 1). In contrast, mRNA and protein levels of fibulin-1, -4, and -5 did not change, after 24 hours of simvastatin treatment (S1 and S2 Tables).

### Mevalonate reverses simvastatin-induced fibulin 2 expression

**Fig 2:**
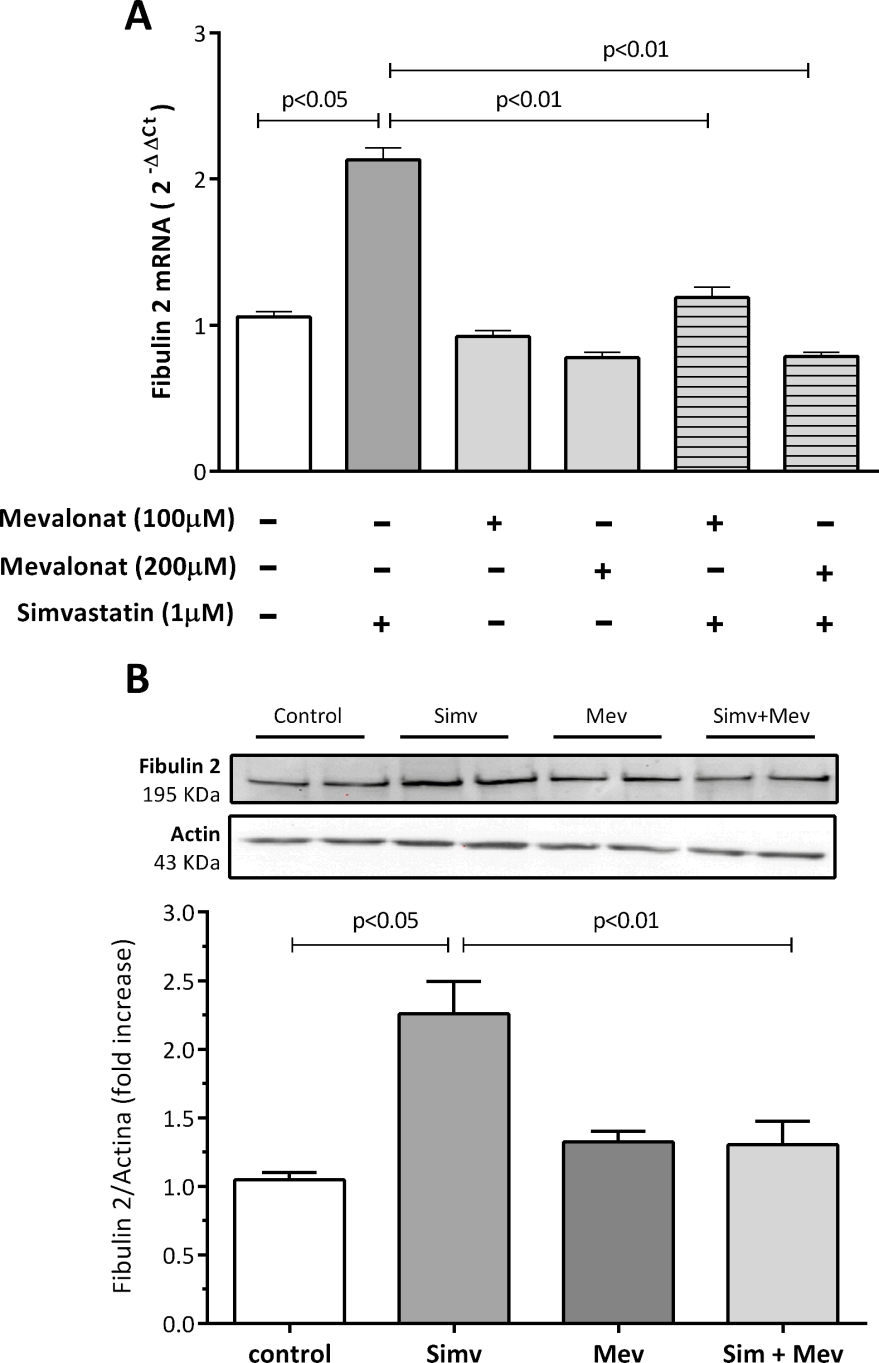
A: Effect of mevalonate on simvastatin-induced fibulin-2 mRNA levels in human coronary artery SMCs. Cells were preincubated for 2 hours with mevalonate (100, 200 μM) and treated with simvastatin for 24 hours. B: Effect of mevalonate on simvastatin-induced fibulin-2 protein levels in human coronary artery SMCs. Cells were preincubated for 2 hours with mevalonate (200 μM) and treated with simvastatin for 24 hours. Blot bands were normalized to actin and quantified with Quantity One Analysis Software version 4.6.2. A representative Western blot is shown. The results are shown as the mean with the standard error of the mean (SEM) for twelve independent experiments in 2A (2 with cell lot number 4C0915, 4 with cell lot number 4C1284, and 6 with cell lot number 886619) and six independent experiments in 2B (2 with cell lot number 4C0915, 2 with cell lot number 4C1284, and 2 with cell lot number 886619). Comparisons were performed using ANOVA followed by Dunett post-test correction.

By inhibiting HMG-CoA reductase, statins cause a depletion of mevalonate in the cells. To determine whether this depletion was involved in simvastatin increase of fibulin 2 expression, we incubated human coronary artery SMCs with simvastatin alone (1 μM) or in combination with mevalonate. We found that pre-incubating the cells for 2 hours with increasing concentrations of mevalonate (100, 200 μM), completely and significantly reversed the simvastatin-dependent induction of fibulin 2 mRNA and protein (Fig 2), confirming the specificity of simvastatin’s effect. Mevalonate alone did not affect fibulin 2 mRNA or protein expression. Furthermore, we showed that mevalonate alone or in combination with simvastatin did not affect fibulin -1, -4, or -5 gene expression (S2 Fig).

### Effect of simvastatin on secreted fibulin-2 expression

**Fig 3:**
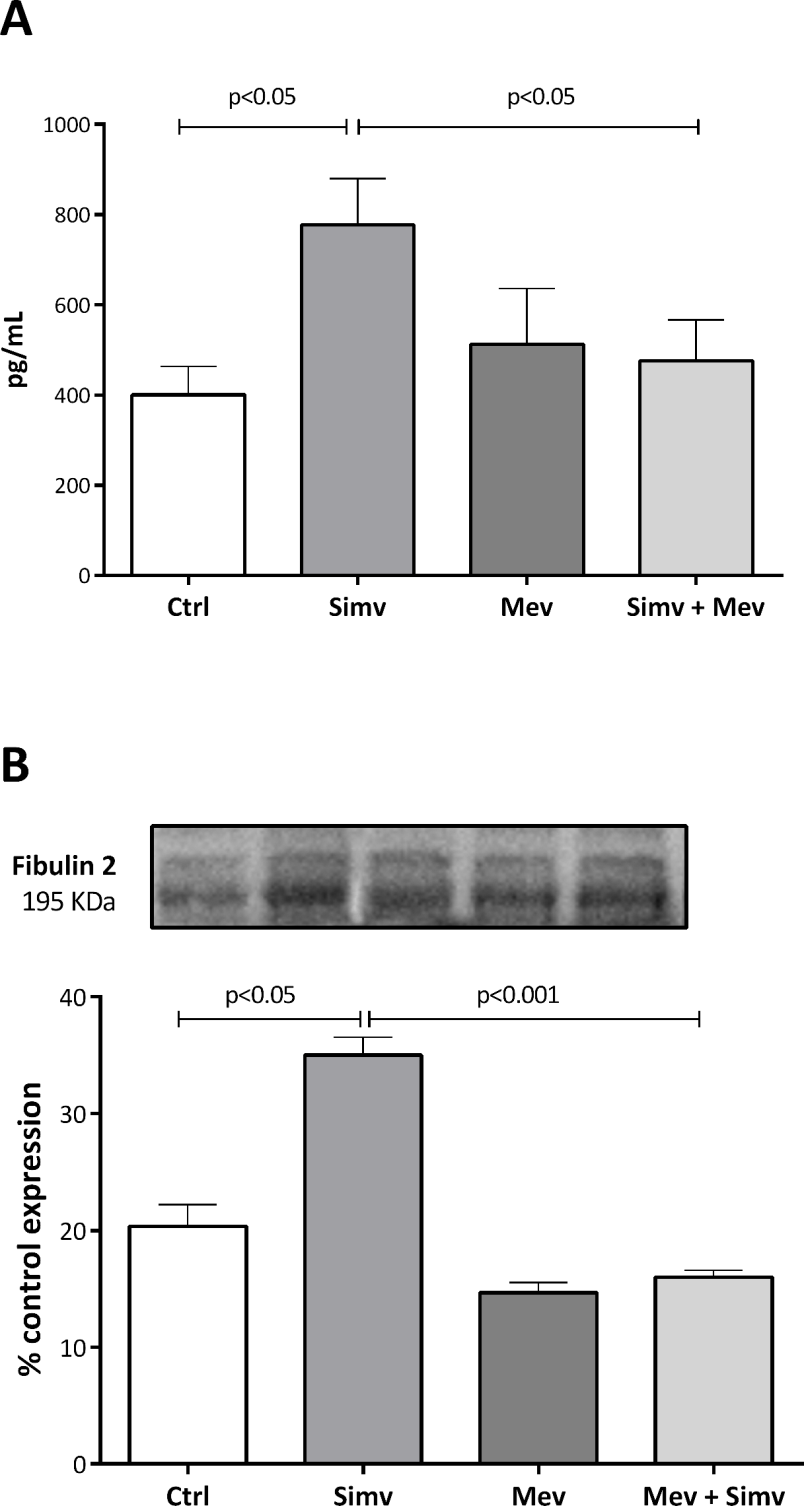
Effect of simvastatin and mevalonate in secreted fibulin 2 expression in culture medium from human coronary artery SMCs. Cells were preincubated for 2 hours with mevalonate (200 μM) and treated with simvastatin for 24 hours. Blot bands were normalized to actin and quantified with Quantity One Analysis Software version 4.6.2. A representative Western blot is shown. The results are shown as the mean with the standard error of the mean (SEM) for four independent experiments in 3A (1 with cell lot number 4C1284 and 3 with cell lot number 886619) and three independent experiments in 2B with cell lot number 886619. Comparisons were performed using ANOVA followed by Dunett post-test correction.

To determine whether the intracellular effects described above for fibulin-2 were valid for secreted fibulin-2, the culture media of those experiments were analyzed by ELISA and western blotting. We found that simvastatin (1 μM) significantly increased secreted fibulin-2 concentration (Fig 3) after 24 h of incubation and that pre-incubating the cells with mevalonate (200 μM), completely and significantly reversed the simvastatin-dependent induction of secreted fibulin protein (Fig 3). These results confirm the specificity of the effect of simvastatin and provide evidence of the secretion of fibulin-2 by these cells. Mevalonate alone did not affect soluble fibulin 2 expression.

### Downstream isoprenoids are involved in simvastatin effect

**Fig 4:**
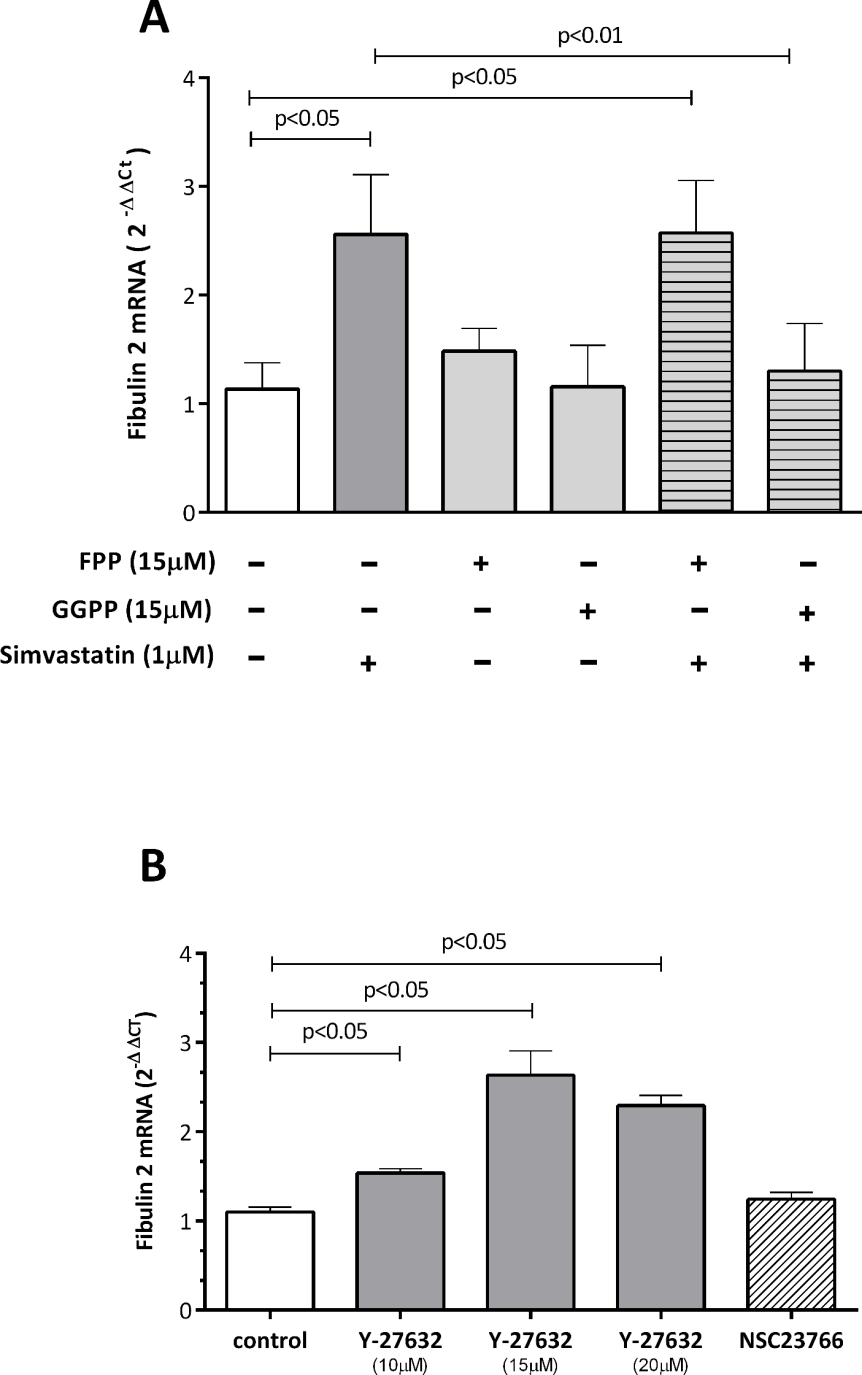
Effect of GGPP and FPP (A) and the ROCK inhibitor Y-27632 and Rac inhibitor NCS23766 (B) on simvastatin-induced fibulin-2 mRNA levels in human coronary artery SMCs. Cells were incubated with simvastatin and isoprenoids or with the inhibitors for 24 hours. The results are shown as the mean with the standard error of the mean (SEM) for twelve independent experiments in both A and B (2 with cell lot number 4C0915, 4 with cell lot number 4C1284, and 6 with cell lot number 886619). Comparisons were performed using ANOVA followed by Dunett post-test correction. FPP: farnesyl pyrophosphate, GGPP: geranylgeranyl pyrophosphate.

Mevalonate is a precursor of isoprenoid compounds such as FPP and GGPP in the cholesterol biosynthetic pathway. To evaluate which downstream isoprenoid was involved in fibulin 2 mRNA expression, we incubated human coronary artery SMCs with simvastatin alone or in combination with one of the isoprenoids. We found that GGPP (15 μM) but not FPP (15 μM) markedly reversed simvastatin-induced fibulin 2 mRNA expression, whereas the respective isoprenoids alone had no effect (Fig 4A). These results were specific for fibulin 2 because no change in the mRNA levels of fibulin-1, -4, or -5 was observed after incubation of cells with isoprenoids alone or in combination with simvastatin (S3 Fig).

### ROCK inhibitor Y-27632 upregulates fibulin 2 expression

Because ROCK is one of the major downstream targets of RhoA, we next evaluated in human coronary artery SMCs the involvement of the RhoA/ROCK pathway on simvastatin-induced fibulin-2 expression. As shown in Fig 4B, treatment of cells with the selective ROCK inhibitor Y-27632 significantly increased fibulin-2 mRNA levels. We found that 24 h treatment with Y-27632 at 10, 15 and 20 μM significantly increased fibulin-2 mRNA expression by 1.5, 2.6, and 2.3-fold, respectively. In contrast, treatment with the selective Rac-1 inhibitor NSC23766 had no effect on fibulin-2 mRNA levels (Fig 4B). Again, this effect was specific for fibulin-2 because incubation of cells with different concentrations of the ROCK inhibitor did not affect the mRNA levels of any of the other fibulins tested (fibulin-1, -4, and -5) (S4 Fig).

### Arachidonic acid, but not other FAs, decreases fibulin-2 gene expression

**Fig 5:**
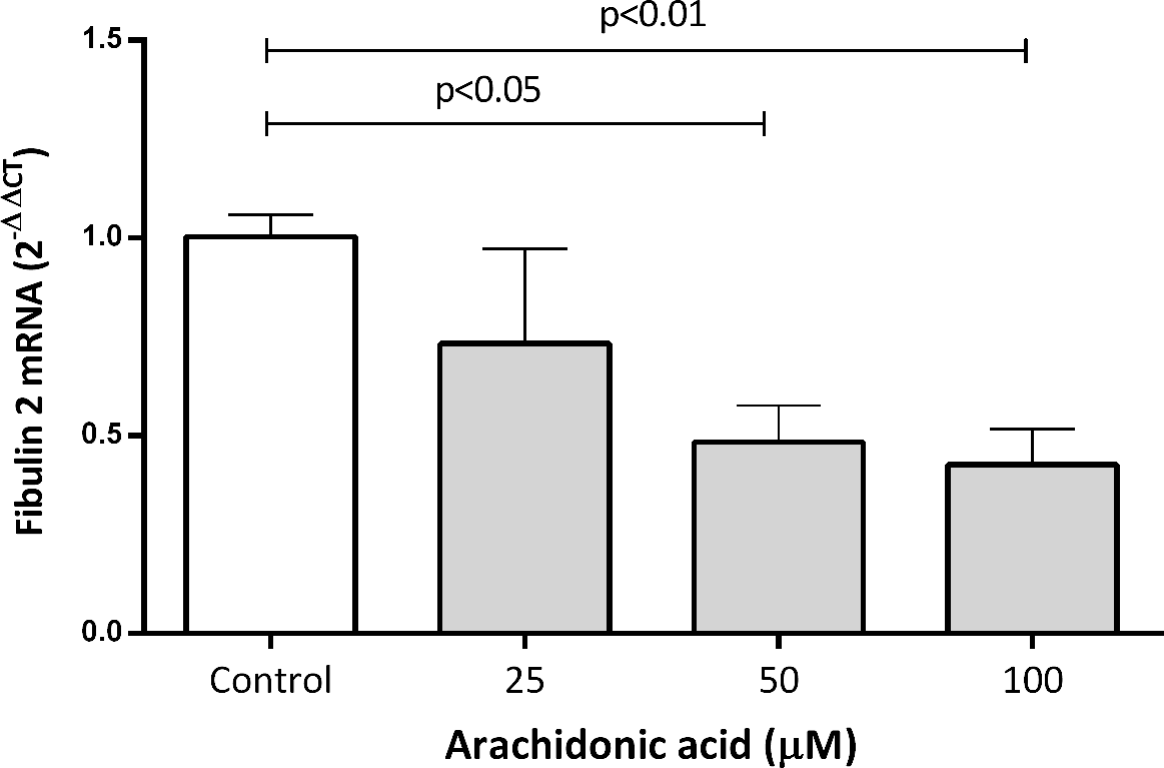
Effect of AA on fibulin-2 mRNA levels in human coronary artery SMCs. Cells were treated with AA for 24 hours. The results are shown as the mean with the standard error of the mean (SEM) for three independent experiments (2 with cell lot number 4C0915 and 1 with cell lot number 4C1284). Comparisons were performed using ANOVA followed by Dunett post-test correction. AA: arachidonic acid.

It is well known that AA, but not PA, OA, LA, EPA or DHA, activates the RhoA/ROCK pathway. Therefore, we tested whether RhoA/ROCK pathway activation with increasing AA concentrations affected fibulin 2 expression. As shown in Fig 5, AA produced a significant decrease in fibulin-2 mRNA levels compared to controls. At 50 and 100 μM, we observed a 45% and 42% decrease, respectively. However, PA, OA, LA, EPA and DHA had no significant effect on fibulin 2 mRNA expression at any of the concentrations used (S5 Fig).

## Discussion

The main objective of the current study was to evaluate whether simvastatin treatment could affect the expression of 4 fibulin family members (fibulin-1, -2, -4, and -5) and the mechanisms involved. We have shown that 24 hours of simvastatin treatment of human coronary artery SMCs upregulates intracellular and secreted fibulin-2 expression at both the mRNA and protein levels. This effect is specific to fibulin-2, as neither fibulin-1, -4 nor -5 were affected by simvastatin treatment. Moreover, we have shown that the mechanism involved is a depletion in the cells of mevalonate and GGPP. Simvastatin by inhibiting the rate-limiting enzyme in the cholesterol synthesis pathway HMG-CoA reductase, decrease levels of these compounds which are intermediates of the pathway. Because GGPP is also responsible for the posttranslational activation of the small GTPase RhoA, the simvastatin-induced depletion of GGPP is linked to an inhibition of RhoA and its effector ROCK. In addition, we observed that the inhibition of ROCK was specific, as treatment with a ROCK inhibitor (Y-27632) increased fibulin-2 expression whereas treatment with a Rac inhibitor (NCS23766) did not. Furthermore, neither mevalonate nor GGPP, FPP, Y-27633, or NSC23766 affect the expression of fibulins -1, -4 or -5. We also found that the induction of RhoA/ROCK pathway with AA decreased fibulin-2 expression, while other fatty acids (PA, OA, LA, EPA and DHA) that don’t induce the pathway, had no effect on fibulin 2 expression. To our knowledge, the simvastatin-induced increase in fibulin-2 expression reported here is a novel non-lipid related effect of simvastatin. Because simvastatin is a lipophilic statin, extrapolation of our results to hydrophilic statins would be just speculation. Therefore, new experiments should be performed with this type of statins to confirm these results.

Statins primarily protect against coronary disease by reducing lipid levels. However, pleiotropic effects of statins can further protect patients on statin therapy. These effects are well characterized and include improvement in endothelial dysfunction, increased nitric oxide bioavailability, antioxidant properties, reduction of blood thrombogenicity, and inhibition of pro-inflammatory responses[29–31].

Fibulin 2 has important features in connection with atherosclerosis. It is an important component of the vascular ECM and can influence the organization and structure of the vascular wall[45–48] by binding to matrix components such as proteoglycans, fibronectin, fibrillin, and laminins [17, 45, 46, 49–51]. Additionally, fibulin 2 can bind to tropoelastin and to fibrillin-1, suggesting that it may act as an anchor for elastin to microfibrils[18, 48, 52]. Moreover, it has been shown in murine models that fibulin-2 is expressed in SMC-rich regions of atherosclerotic lesions, where it colocalizes with versican and hyaluronan[28]. Moreover, it has been shown that thinning of the plaque fibrous cap and the presence of immature intraplaque neovessels are key events in the transformation of atherosclerotic plaques to a vulnerable phenotype, thus contributing to the onset of complications[53]. In this regard, an important pleiotropic effect of statins is the improvement in the characteristics that stabilizes atherosclerotic plaques[37, 39]. For instance, 12-month treatment of patients with atorvastatin has resulted in significant increases in the fibrotic content of the plaque [36]. Immunohistochemistry studies of human carotid plaques have shown that patients on pravastatin have plaques with a significant higher collagen content [35]. Moreover, 9-month statin treatment after acute myocardial infarction significantly increased the fibrous-cap thickness in patients with hyperlipidemia[38]. But fibulin-2 not only is a scaffold protein, there is also evidence that it has a regulatory function because it can modify SMC migration[28, 51]. Moreover, it has been proposed that changes in fibulin-2 structure or levels may partially control systolic blood pressure[54]. Overall, the role of fibulin-2 in the atherosclerotic process needs to be clarified with more studies.

We describe a mechanism that has been widely reported as a mode of action of statins. One example related to the stabilization effect of statins is that statins inhibit MMP-2 and MMP-9 secretion through the suppression of RhoA activation and Rab prenylation, respectively[55, 56]. Statins inhibit the isoprenylation of small G-proteins by inhibiting the synthesis of important isoprenoids, which prevents the attachment of the small G-proteins to the cell membrane, thereby inhibiting their ability to transduce signals through MAPK pathways.

In conclusion, the simvastatin-induced upregulation of fibulin-2 expression may affect the composition and structure of the ECM. Whether this effect has a beneficial impact on atherosclerosis plaque characteristics needs further investigation.

## Supporting Information

S1 Fig Effect of simvastatin on fibulin-1, -2, -4 and -5 mRNA levels in human coronary artery SMCs. Cells were treated with simvastatin for 6 hours. The results are shown as the mean with standard error of the mean for three independent experiments. Comparisons were performed using ANOVA followed by Dunnett post-test correction.
(DOCX)

S2 Fig Effect of mevalonate and simvastatin on fibulin-1, -4, and -5 mRNA levels in human coronary artery SMCs. Cells were pre incubated for 2 hours with mevalonate (100 μM) and treated with simvastatin (1μM) for 24 hours. The results are shown as the mean with the standard deviation for at least three independent experiments. Comparisons were performed using ANOVA followed by Dunett post-test correction.
(DOCX)

S3 Fig Effect of FPP, GGPP, and simvastatin on fibulin-1, -4, and -5 mRNA levels in human coronary artery SMCs. Cells were incubated with simvastatin (1μM) and isoprenoids (15μM) for 24 hours. The results are shown as the mean with the standard deviation for at least three independent experiments. Comparisons were performed using ANOVA followed by Dunett post-test correction. FPP: farnesyl pyrophosphate, GGPP: geranylgeranyl pyrophosphate, Simv: simvastatin.
(DOCX)

S4 Fig Effect of the ROCK inhibitor Y-27632 and Rac inhibitor NCS23766 on fibulin-1, -4, and -5 mRNA levels in human coronary artery SMCs. Cells were incubated with the inhibitors for 24 hours. The results are shown as the mean with the standard deviation for at least three independent experiments. Comparisons were performed using ANOVA followed by Dunett post-test correction.
(DOCX)

S5 Fig Effect of fatty acids on fibulin-2 mRNA levels in human coronary artery SMCs. Cells were treated with different concentrations of fatty acids for 24 hours. The results are shown as the mean with the standard deviation for three independent experiments. Comparisons were performed using ANOVA followed by Dunett post-test correction. PA: Palmitic acid, OA: oleic acid, LA: linoleic acid, EPA: eicosapentaenoic acid, DHA: docosahexaenoic acid.
(DOCX)

S1 Table Effect of simvastatin on fibulin -1, -4, and -5 mRNA levels in human coronary artery SMCs. Cells were treated with different concentrations simvastatin for 24 hours. The results are shown as the mean with standard deviation for three independent experiments. Comparisons were performed using ANOVA followed by Dunnett post-test correction.
(DOCX)

S2 Table Effect of simvastatin on fibulin -1 and -5 protein levels in human coronary artery SMCs. Cells were treated with different concentrations simvastatin for 24 hours. The results are shown as the mean with standard deviation for three independent experiments. Comparisons were performed using ANOVA followed by Dunnett post-test correction.
(DOCX)
