# Supplementary material for: Simvastatin Increases Fibulin-2 Expression in Human Coronary Artery Smooth Muscle Cells via RhoA/Rho-Kinase Signaling Pathway Inhibition
Source: PLoS One. 2015 Jul 24;10(7):e0133875. doi: 10.1371/journal.pone.0133875 (PMC4514789; doi:10.1371/journal.pone.0133875)
Supplement: S1 Table — Cells were treated with different concentrations simvastatin for 24 hours. The results are shown as the mean with standard deviation for three independent experiments. Comparisons were performed using ANOVA followed by Dunnett post-test correction. (DOCX) [file pone.0133875.s006.docx]

|  |  | Fibulin-1 | |  | Fibulin-4 | |  | Fibulin-5 | |
| --- | --- | --- | --- | --- | --- | --- | --- | --- | --- |
|  |  | mRNA (2^-ΔΔCt^) | |  | mRNA (2^-ΔΔCt^) | |  | mRNA (2^-ΔΔCt^) | |
|  |  | mean | SD |  | mean | SD |  | mean | SD |
|  |  |  |  |  |  |  |  |  |  |
|  | control | 1 |  |  | 1 |  |  | 1 |  |
| Simvastatin | 0.05 µM | 0,81 | 0,09 |  | 1,31 | 0,00 |  | 0,87 | 0,17 |
|  | 0.1 µM | 0,81 | 0,13 |  | 1,32 | 0,07 |  | 1,21 | 0,13 |
|  | 0.5 µM | 0,87 | 0,39 |  | 1,39 | 0,33 |  | 0,92 | 0,01 |
|  | 1 µM | 0,74 | 0,08 |  | 0,92 | 0,05 |  | 0,91 | 0,18 |
