# Supplementary material for: Simvastatin Increases Fibulin-2 Expression in Human Coronary Artery Smooth Muscle Cells via RhoA/Rho-Kinase Signaling Pathway Inhibition
Source: PLoS One. 2015 Jul 24;10(7):e0133875. doi: 10.1371/journal.pone.0133875 (PMC4514789; doi:10.1371/journal.pone.0133875)
Supplement: Supplementary file 3 [file pone.0133875.s001.docx]

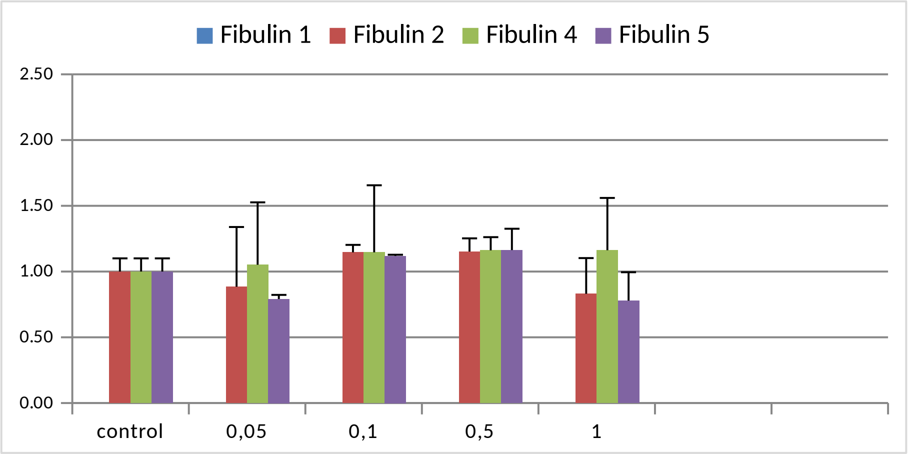
Fibulin 1
Fibulin 2
Fibulin 4
Fibulin 5
2.50
2.00
1.50
1.00
0.50
0.00
control
0,05
0,1
0,5
1
