# Supplementary material for: Simvastatin Increases Fibulin-2 Expression in Human Coronary Artery Smooth Muscle Cells via RhoA/Rho-Kinase Signaling Pathway Inhibition
Source: PLoS One. 2015 Jul 24;10(7):e0133875. doi: 10.1371/journal.pone.0133875 (PMC4514789; doi:10.1371/journal.pone.0133875)
Supplement: Supplementary file 4 [file pone.0133875.s002.docx]

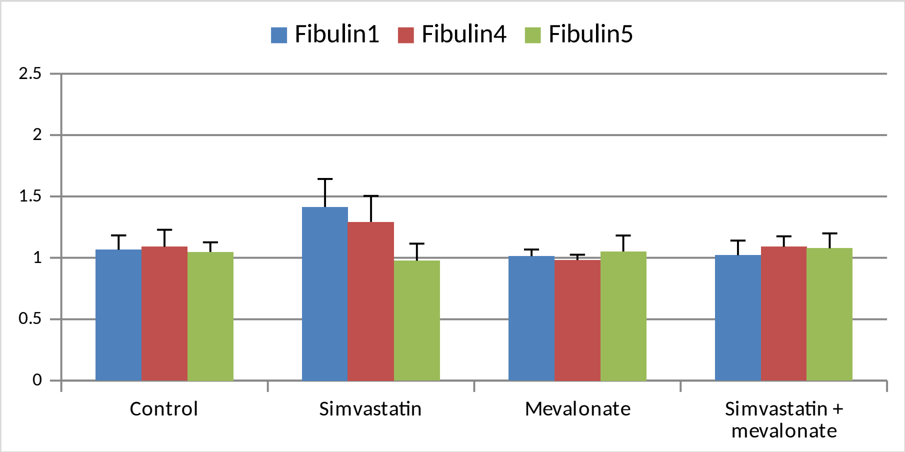

Fibulin1
Fibulin4
Fibulin5
2.5
2
1.5
1
0.5
0
Control
Simvastatin
Mevalonate
Simvastatin + mevalonate
