# Supplementary material for: Simvastatin Increases Fibulin-2 Expression in Human Coronary Artery Smooth Muscle Cells via RhoA/Rho-Kinase Signaling Pathway Inhibition
Source: PLoS One. 2015 Jul 24;10(7):e0133875. doi: 10.1371/journal.pone.0133875 (PMC4514789; doi:10.1371/journal.pone.0133875)
Supplement: Supplementary file 6 [file pone.0133875.s004.docx]

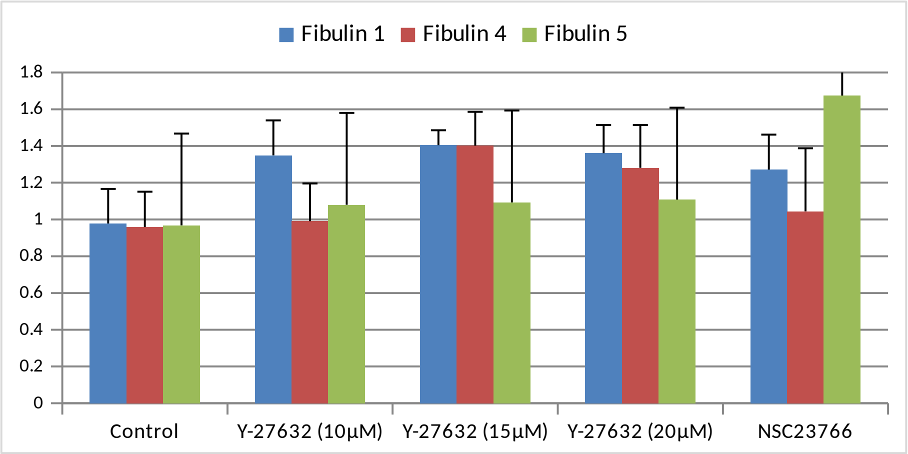
Fibulin 1
Fibulin 4
Fibulin 5
1.8
1.6
1.4
1.2
1
0.8
0.6
0.4
0.2
0
Control
Y-27632 (10µM)
Y-27632 (15µM)
Y-27632 (20µM)
NSC23766
