# Supplementary material for: Simvastatin Increases Fibulin-2 Expression in Human Coronary Artery Smooth Muscle Cells via RhoA/Rho-Kinase Signaling Pathway Inhibition
Source: PLoS One. 2015 Jul 24;10(7):e0133875. doi: 10.1371/journal.pone.0133875 (PMC4514789; doi:10.1371/journal.pone.0133875)
Supplement: Supplementary file 7 [file pone.0133875.s005.docx]

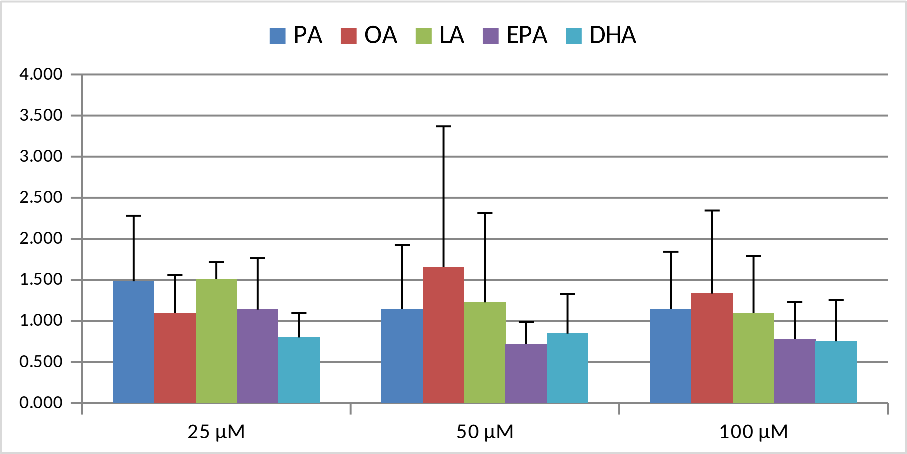
PA
OA
LA
EPA
DHA
4.000
3.500
3.000
2.500
2.000
1.500
1.000
0.500
0.000
25 µM
50 µM
100 µM
